# Neuregulin-4 Is Required for the Growth and Elaboration of Striatal Medium Spiny Neuron Dendrites

**DOI:** 10.1093/jnen/nlz046

**Published:** 2019-05-24

**Authors:** Blanca Paramo, Sean Wyatt, Alun M Davies

**Affiliations:** School of Biosciences, Cardiff University, Cardiff, UK

**Keywords:** Dendrites, Medium spiny neurons, Neuregulin-4, Striatum

## Abstract

Medium spiny neurons (MSNs) comprise the vast majority of neurons in the striatum. Changes in the exuberant dendrites of these widely connected neurons are associated with a multitude of neurological conditions and are caused by a variety of recreational and medicinal drugs. However, we have a poor understanding of the physiological regulators of dendrite growth and elaboration of this clinically important population of neurons. Here, we show that MSN dendrites are markedly smaller and less branched in neonatal mice that possess a homozygous null mutation in the neuregulin-4 gene (*Nrg4^−^*^/^^*−*^) compared with wild type (*Nrg4*^+/+^) littermates. *Nrg4^−^*^/^^*−*^ mice also had a highly significant reduction in MSN dendrite spine number in neonates and adults. The striking stunted dendrite arbor phenotype of MSNs observed in *Nrg4^−^*^/^^*−*^ neonates was replicated in MSNs cultured from *Nrg4^−^*^/^^*−*^ embryos and was completely rescued by soluble recombinant neuregulin-4. MSNs cultured from wild type mice coexpressed NRG4 and its receptor ErbB4. Our findings show that NRG4 is a major novel regulator of dendritic growth and arborization and spine formation in the striatum and suggest that it exerts its effects by an autocrine/paracrine mechanism.

## INTRODUCTION

Medium spiny neurons (MSNs) comprise 90%–95% of the neurons in the striatum. These GABAergic inhibitory neurons have exuberant dendritic arbors that are densely studded with dendritic spines. They receive large numbers of afferents from major brain regions, including extensive glutaminergic input from the cortex, hippocampus, amygdala, and thalamus and dopaminergic input from the ventral tegmental area and substantia nigra (1). MSNs are projection neurons whose axons terminate in the substantia nigra and globus pallidus (2). Because of their extensive and unique connections, MSNs play a central role in the control of voluntary movement, procedural memory and motivated behavior (3). Degeneration and loss of MSNs is the pathognomonic feature of Huntington disease and underlies the symptomology of this hereditary neurodegenerative disorder (4). In addition to Huntington disease, dendritic changes in MSNs are associated with a range of neurological disorders, including Parkinson disease, dystonia, compulsivity, addiction, hyperactivity, and depression (2, 5–9). Because dendrite morphology and spine density are key determinants of the functional properties of neurons and neural circuits in health and disease, the changes in MSN dendrites in so many neurological disorders emphasize the need to understand the physiological regulators of MSN dendrite morphology and the formation of dendrite spines.

While administration of drugs, such as nicotine, cocaine, and neurotransmitter receptor agonists and antagonists, to rodents is associated with changes in MSN dendrite morphology and spine density (7, 10), the clearest evidence for physiologically relevance of particular proteins controlling striatal MSN dendrite morphology has come from studies of transgenic manipulation of the expression of particular proteins. For example, decreased MSN spine density has been reported in mice that are heterozygous for a null mutation in *bdnf* gene (11) and in mice that lack the diacylglycerol kinase-β gene (12). Decreased MSN dendritic arborization has been reported in mice that overexpress dopamine D2 receptors in MSNs (13). Conversely, increased MSN dendritic arborization has been reported in mice that lack the *Cd40* gene, and in vitro experiments suggest that CD40-activated CD40L reverse signaling suppresses MSN dendrite growth and elaboration by a PKCγ-dependent mechanism (14).

Here, we have investigated the role of neuregulin-4 (NRG4) in regulating the growth and elaboration of MSN dendrites and dendrite spine formation in developing mice. NRG4 is one of the neuregulins that are widely expressed pleiotropic growth factors related to epidermal growth factor that signal via the ErbB family of receptor tyrosine kinases (15). The neuregulins, especially the numerous isoforms of NRG1, have numerous roles in the development and function of neurons and glia, including regulating the assembly of neural circuitry, myelination, neurotransmission, and synaptic plasticity (16). Importantly, the *Nrg1*, *Nrg2*, *Nrg3*, *ErbB3*, and *ErbB4* genes have been identified as susceptibility genes for schizophrenia, depression, and bipolar disorder (16, 17) and numerous genetic and functional studies have directly implicated the *Nrg1*, *Nrg2*, *Nrg3*, and *ErbB4* genes in the development of psychotic behavior (18–22). Unlike other neuregulins, NRG4 is expressed at much lower levels in the brain, and only one function has been described for this factor in the brain so far. The dendrites of neocortical pyramidal neurons of *Nrg4*^*−*^^/^^*−*^ mice are stunted compared with wild type mice, suggesting that NRG4 regulates dendritic arborization in the developing cerebral cortex (23). In the present study, we have compared the striatal MSNs of *Nrg4*^+/+^ and *Nrg4*^*−*^^/^^*−*^ mice. We show that striatal MSN dendrites are markedly smaller and less branched in NRG4-deficient mice compared with those of wild type mice in vivo and in vitro and have a greatly reduced number of dendritic spines, suggesting that NRG4 plays a major novel role in dendrite development from this clinically important population of neurons.

## MATERIALS AND METHODS

### Animals

Mice were housed in a 12-hour light-dark cycle with access to food and water ad libitum. Breeding was approved by the Cardiff University Ethical Review Board and was performed within the guidelines of the Home Office Animals (Scientific Procedures) Act, 1986. *Nrg4* null mice in which the *Nrg4* locus was disrupted by retroviral insertion of a gene trap between exons 1 and 2 were purchased from the Mutant Mouse Resource Center, UC Davis (Davis, CA). These mice were backcrossed from a C57/BL6 background into a CD1 background. *Nrg4*^+/^^*−*^ mice were crossed to generate *Nrg4*^+/+^ and *Nrg4*^*−*^^/^^*−*^ littermates.

### Neuron Culture

MSN cultures were prepared from E14 striatal primordia that were triturated to produce a single cell suspension following trypsin digestion (Worthington, Lakewood, NJ) and DNase I treatment (Roche Applied Science, Burgess Hill, UK). To increase the yield of MSN and avoid contamination with other regions, striata were thoroughly cleaned after dissection and cells were grown in a media that promotes neuronal growth while preventing the growth of other nondifferentiated cells. Neurons were plated at a density of 15 000 cells/cm^2^ at the center of 35-mm dishes coated with poly-l-lysine (Sigma-Aldrich, Gillingham, UK). Cells were cultured with Neurobasal A (Invitrogen, Paisley, UK) supplemented with 2% B27 (Invitrogen), 1% fetal calf serum (Sigma-Aldrich), 100 units/mL penicillin and 100 μg/mL streptomycin (Gibco BRL, UK). To avoid extensive astrocyte proliferation, the medium was changed to medium without fetal calf serum after 7 days in vitro. The cultures were incubated at 37°C in a humidified atmosphere containing 5% CO_2_ and were treated with recombinant human NRG4 (Thermo Fisher Scientific) the same day after plating, as indicated. No other growth factors were added to the medium. The culture medium was partially replaced with fresh medium with the treatments after 4–5 days. The cultures were fixed 10 or 12 days after plating.

### Immunocytochemistry

After 10–12-day in vitro, neurons were fixed for 10 minutes in 4% paraformaldehyde in 0.12 M phosphate-buffered saline (PBS), washed 3 times in PBS, and blocked in 1% bovine serum albumin (Sigma), 0.1% Triton (Sigma) in PBS for 1 hour, then incubated with primary antibodies against either NRG4 (1:200, goat-polyclonal, Santa Cruz), ErbB4 (1:200, rabbit or mouse-monoclonal, Abcam, Cambridge, MA), DARPP-32 (1:400, rabbit-monoclonal, Cell Signaling Technology, Danvers, MA) or βIII-tubulin (1:500, mouse-monoclonal, R&D Systems) at 4°C overnight. After washing, the neurons were incubated with polyclonal Alexa-conjugated secondary antibodies (donkey anti-goat Alexa-546, donkey anti-rabbit Alexa-488, Invitrogen) 1:500 for 1 hour at room temperature. Cells were then washed, incubated with DAPI (1:8000) and visualized using a Zeiss LSM710 confocal microscope. For double immunolabeling, the cultures were incubated simultaneously with both primary and secondary antibodies.

### Quantification of Dendrite Morphology In Vitro

Total neurite length, the number of branching points and Sholl analysis was performed on DARPP-32-positive neurons. The soma was labeled with anti-βIII-tubulin and the length was quantified using FIJI (Image J) as previously described (23).

### Immunohistochemistry

Brains were fixed overnight using 4% paraformaldehyde in 0.12 M PBS at 4°C, washed in PBS and cryoprotected in 30% sucrose before being frozen in dry ice-cooled isopentane. Serial 30 μm sections were blocked in 1% bovine serum albumin (Sigma), 0.1% Triton (Sigma) in PBS and then incubated with 1:400 rabbit-monoclonal anti-DARPP32 (Cell Signaling) and 1:200 goat-polyclonal anti-NRG4 (Santa Cruz Biotechnologies, Santa Cruz, CA) antibodies at 4°C overnight. After washing, the sections were incubated with 1:500 of donkey anti-rabbit or goat-polyclonal Alexa-488 or Alexa-647 conjugated secondary antibodies (Invitrogen) for 1 hour at room temperature. Sections were washed, incubated with DAPI, and visualized using a Zeiss LSM710 confocal microscope.

### Quantitative PCR

The levels of *Nrg4* and *ErbB4* mRNAs in the striatum were quantified by RT-qPCR relative to a geometric mean of mRNAs for the house keeping enzymes glyceraldehyde phosphate dehydrogenase (*Gapdh* mRNA), succinate dehydrogenase (*Sdha* mRNA), and hypoxanthine phosphoribosyltransferase-1 (*Hprt1* mRNA). Total RNA was extracted from dissected striata with the RNeasy Mini Lipid extraction kit (Qiagen, Crawely, UK). Five microliters of total RNA was reverse transcribed, for 1 hour at 45°C, using the AffinityScript kit (Agilent, Berkshire, UK) in a 25 µL reaction according to the manufacturer’s instructions. Two microliters of cDNA was amplified in a 20 µL reaction volume using Brilliant III ultrafast qPCR master mix reagents (Agilent Technologies). PCR products were detected using dual-labeled (FAM/BHQ1) hybridization probes specific to each of the cDNAs (MWG/Eurofins, Ebersberg, Germany). The PCR primers were *Nrg4* forward: 5′-GAG ACA AAC AAT ACC AGA AC-3′ and reverse: 5′-GGA CTG CCA TAG AAA TGA-3′; *ErbB4* forward: 5′-GGC AAT ATC TAC ATC ACT G-3′ and reverse: 5′-CCA ACA ACC ATC ATT TGA A-3′; *Gapdh* forward: 5′-GAG AAA CCT GCC AAG TAT G-3′ and reverse: 5′-GGA GTT GCT GTT GAA GTC-3′; *Sdha* forward: 5′-GGA ACA CTC CAA AAA CAG-3′ and reverse: 5′-CCA CAG CAT CAA ATT CAT-3′; and *Hprt1* forward: 5′-TTA AGC AGT ACA GCC CCA AAA TG-3′ and reverse: 5′-AAG TCT GGC CTG TAT CCA ACA C-3′. Dual-labeled probes were *Nrg4*: 5′-FAM-CGT CAC AGC CAC AGA GAA CAC-BHQ1-3′; *ErbB4*: 5′-FAM-AGC AAC CTG TGT TAT TAC CAT ACC ATT-BHQ1-3′; *Gapdh*: 5′-FAM-AGA CAA CCT GGT CCT CAG TGT-BHQ1-3; *Sdha*: 5′-FAM-CCT GCG GCT TTC ACT TCT CT-BHQ1-3; and *Hrpt1*: 5′-FAM-TCG AGA GGT CCT TTT CAC CAG CAA G-BHQ1-3′. Forward and reverse primers were used at a concentration of 250 nM and dual-labeled probes were used at a concentration of 500 nM. PCR was performed using the Mx3000P platform (Agilent) using the following conditions: 95°C for 3 minutes followed by 45 cycles of 95°C for 10 seconds and 60°C for 35 seconds. Standard curves were generated for each cDNA for every real time PCR run, by using serial 3-fold dilutions of reverse transcribed adult mouse brain total RNA (Zyagen, San Diego, CA). Relative mRNA levels were quantified in the striatum dissected from 4 animals at each age. Primer and probe sequences were designed using Beacon Designer software (Premier Biosoft, Palo Alto, CA).

### Golgi Staining

Modified Golgi-Cox impregnation of neurons was performed using the FD Rapid GolgiStain kit (FD NeuroTechnologies, Columbia, MD) according to the manufacturer’s instructions on 100 μm sagittal sections of P9, juvenile (P30), and adult (P60) mouse brains of *Nrg4*^+/+^ and *Nrg4*^*−*^^/^^*−*^ littermates. Total dendrite length, branch point number and Sholl analysis was carried out using the plugin Sholl Analysis of Fiji software (24) after neuronal reconstruction with the plugin Simple Neurite Tracer (25). The number of spines in 100 μm dendrite lengths chosen at random was counted and each dendritic spine was classified on the basis of accepted morphology into 1 of 2 types: immature (either filopolial or thin) and mature (either stubby, mushroom or branched) (26).

## RESULTS

### Expression of *Nrg4* and *ErbB4* Transcripts in the Developing Striatum

**FIGURE 1.**
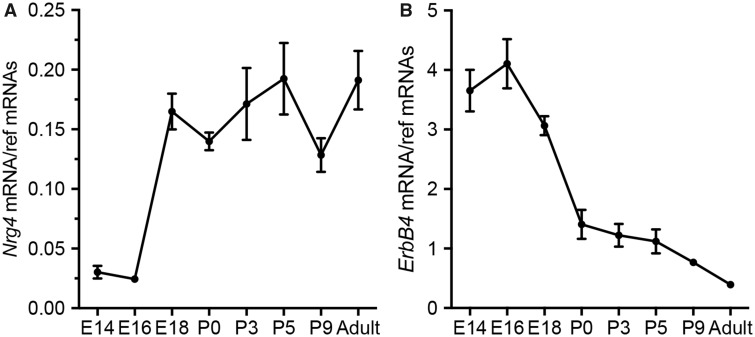
Developmental time course of *Nrg4* and *ErbB4* mRNA expression in the developing striatum. Levels of *Nrg4* mRNA **(A)** and *ErbB4* mRNA **(B)** in the striatum of wild type mice at different ages relative to the geometric mean of reference mRNAs (*Gapdh*, *Sdha*, and *Hprt1* mRNAs). The mean ± SEM of data from 4 separate sets of dissected striatum at each age are plotted.

To begin to explore the possibility that NRG4 functions in the developing striatum, we used qPCR to investigate if transcripts encoding NRG4 and ErbB4, which is a receptor for most neuregulins, including NRG4, are present in the striatum dissected at different ages. This revealed that although *Nrg4* mRNA and *ErbB4* mRNA were detectable in the embryonic and postnatal striatum at all stages studied, the expression profiles differed. Whereas *Nrg4* mRNA displayed a large increase after E16 and plateaued from E18 onward, *ErbB4* mRNA expression peaked at E16 and decreased with age (Fig. 1).

### Striatal MSN Dendrites Are Markedly Stunted in Neonatal *Nrg4*^*−*^^/^^*−*^ Mice

**FIGURE 2.**
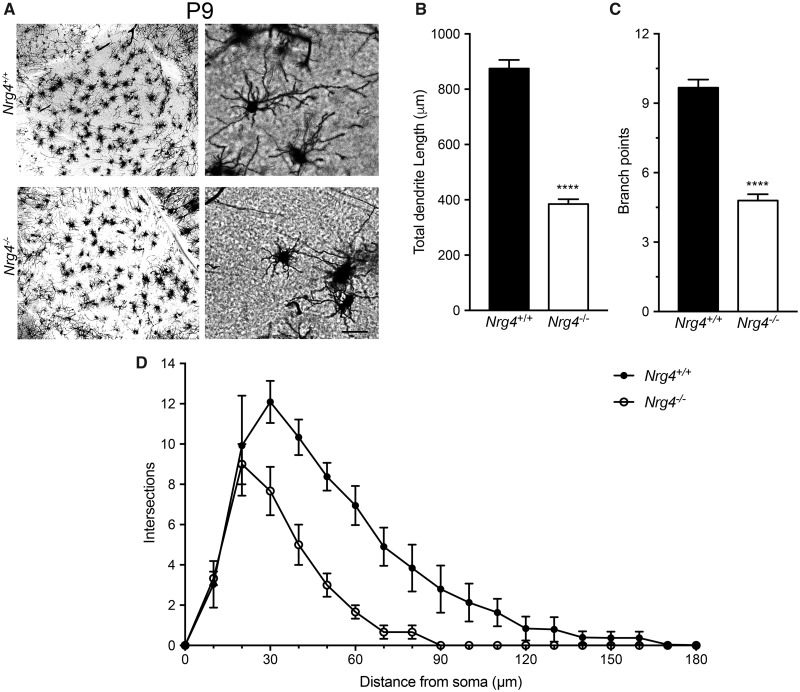
Phenotypic changes in MSN dendrites of *Nrg4^−^*^/^^*−*^ mice at P9. **(A)** Representative low-power images of the striatum and high-power images of striatal MSNs in Golgi preparations of *Nrg4*^+/+^ and *Nrg4^−^*^/^^*−*^ mice. Scale bar = 50 μm. Quantification of total dendrite length **(B)** and number of branch points in the dendrites **(C)** of striatal MSNs of P9 *Nrg4*^+/+^ and *Nrg4^−^*^/^^*−*^ littermates **(D)**. Sholl plots of the dendrites of these neurons. The mean ± SEM of data from 90 neurons (from at least 3 different animals per genotype) are plotted (****p < 0.0001, statistical comparison between *Nrg4*^+/+^ and *Nrg4^−^*^/^^*−*^ mice, 2-tailed *t*-test).

To investigate the significance of *Ngr4* mRNA expression in the developing striatum, we compared *Nrg4*^*−*^^/^^*−*^ and *Nrg4*^+/+^ mice. Golgi preparations of the striatum were initially examined in postnatal day 9 (P9) mice. These revealed that the size and complexity of the dendritic arbors of MSNs were dramatically reduced throughout the striatum in *Nrg4*^*−*^^/^^*−*^ mice compared with *Nrg4*^+/+^ littermates (Fig. 2A). Quantitative analysis carried out on the dendrite compartments revealed highly significant 2-fold reductions in total dendrite length (Fig. 2B) and branch point number (Fig. 2C). These reductions in dendrite length and branching were reflected in the Sholl analyses of the dendrite arbors (Fig. 2D). These findings suggest that NRG4 plays a major role in promoting the growth and elaboration of MSN dendrites in the developing striatum.

**FIGURE 3.**
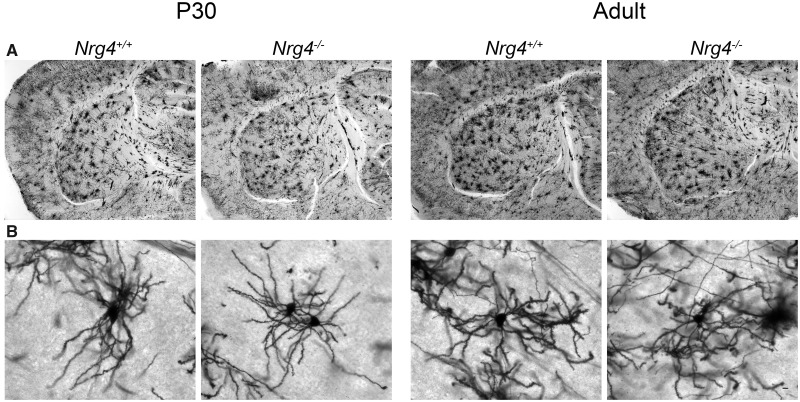
MSNs of juvenile (P30) and adult (P60) *Nrg4*^+/+^ and *Nrg4^−^*^/^^*−*^ mice. Representative low-power images of the striatum **(A)** and high-power images of MSNs **(B)** of Golgi preparations of *Nrg4*^+/+^ and *Nrg4^−^*^/^^*−*^ mice. Scale bars = 50 μm for **A** and 10 μm for **B**. Samples from at least 3 animals per genotype were analyzed.

To determine whether the pronounced phenotype observed in neonatal *Nrg4*^*−*^^/^^*−*^ mice is retained into adulthood, we repeated the Golgi studies in juvenile (P30) and adult (P60) *Nrg4*^+/+^ and *Nrg4*^*−*^^/^^*−*^ littermates. In contrast to the clear and quantifiable differences observed in the striatal MSN dendrites in postnatal *Nrg4*^+/+^ and *Nrg4*^*−*^^/^^*−*^ mice, differences in the size and complexity of these dendrites were not clearly discernable at later ages (Fig. 3A, B). Unfortunately, the large size and complexity of MSN dendrites in juvenile and adult mice and the fact that many of the dendritic arbors were overlapping in our Golgi preparations precluded accurate quantification.

### Decreased Number of MSN Dendrite Spines in *Nrg4*^*−*^^/^^*−*^ Mice

**FIGURE 4.**
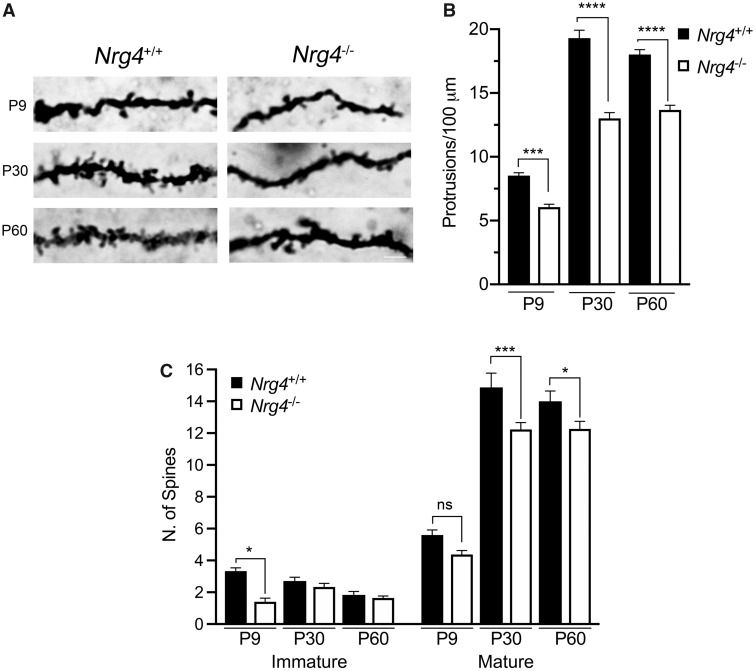
Reduction in MSN dendrite spine density in *Nrg4^−^*^/^^*−*^ mice. **(A)** Representative images showing the dendritic spines of *Nrg4*^+/+^ and *Nrg4^−^*^/^^*−*^ mice at P9, P30, and P60. Scale bar = 10 μm. **(B)** Quantification of the total number of spines at different ages from *Nrg4*^+/+^ and *Nrg4^−^*^/^^*−*^ mice. Mean of total number ± SEM from 150 independent 100 μm segments from different neurons per genotype (***p < 0.001 and ****p < 0.0001, one-way ANOVA with Tukey’s multiple comparison post hoc test). **(C)** Quantification of immature (filopodia and long thin) and mature (stubby, mushroom and branched) spines of dendrites of *Nrg4*^+/+^ and *Nrg4^−^*^/^^*−*^ mice at P9, P30, and P60 (ns, not significant, *p < 0.05, ***p < 0.001, one-way ANOVA with Fisher’s multiple comparison post hoc test).

Because of the difficulty of quantifying the size and complexity of MSN dendrite arbors in older mice, we examined another parameter of MSN dendrites that can be easily observed and quantified in developing and mature mice, namely, dendrite spine number per unit dendrite length and dendrite spine morphology. High-resolution images of MSN dendrite spines in Golgi preparations revealed a reduction in dendrite spine number per unit dendrite length in *Nrg4*^*−*^^/^^*−*^ mice compared with and *Nrg4*^+/+^ mice at P9, P30 and P60 (Fig. 4A). Quantification of the number spines in 100 μm lengths of MSN dendrites chosen at random revealed highly significant reductions in *Nrg4*^*−*^^/^^*−*^ mice compared with *Nrg4*^+/+^ mice at all 3 ages (Fig. 4B). The magnitude of the reduction was similar at each age (∼30% reduction), suggesting that this reduction arises in development and is sustained in the adult. Because of the reduced total length of MSN dendrite arbors quantified in neonatal *Nrg4*^*−*^^/^^*−*^ mice (Fig. 2), the highly significant reduction in MSN spine number per unit length in *Nrg4*^*−*^^/^^*−*^ mice implies an even greater decrease in the total number of dendrite spines on MSNs in *Nrg4*^*−*^^/^^*−*^ neonates.

In addition to counting dendritic spine number, we also classified each spine into immature or mature types depending on generally accepted morphological criteria (26). While there were marked increases in the number of mature spines in both genotypes between P9 and P30, there were significantly fewer mature spines in *Nrg4*^*−*^^/^^*−*^ mice compared with and *Nrg4*^+/+^ mice at P30 and P60 (Fig. 4C). Although there were fewer immature spines than mature spines at all ages in both genotypes, there were significantly more immature spines in *Nrg4*^+/+^ mice than *Nrg4*^*−*^^/^^*−*^ mice at P9 just before the marked increase in the number of mature spines (Fig. 4C).

### MSNs Cultured from *Nrg4*^*−*^^/^^*−*^ Embryos Have Stunted Neurites

To ascertain whether the stunted dendrite phenotype observed in developing *Nrg4*^*−*^^/^^*−*^ mice in vivo is replicated in vitro and can be rescued by NRG4 treatment, we set up dissociated MSN cultures from *Nrg4*^*−*^^/^^*−*^ and *Nrg4*^+/+^ littermates. Cultures were established from E14 striatal primordial and were fixed and examined 10 days after plating. MSNs were positively identified using an antibody to dopamine and cyclic AMP-regulated protein (DARPP-32), which is expressed by more than 95% of MSNs (27). Cultures were double labeled with anti-DARPP-32 and anti-βIII tubulin, which labels all neurons. Quantification showed that 69.8% ± 1.2% (mean ± SEM) of the cells were positive for DARPP-32.

**FIGURE 5.**
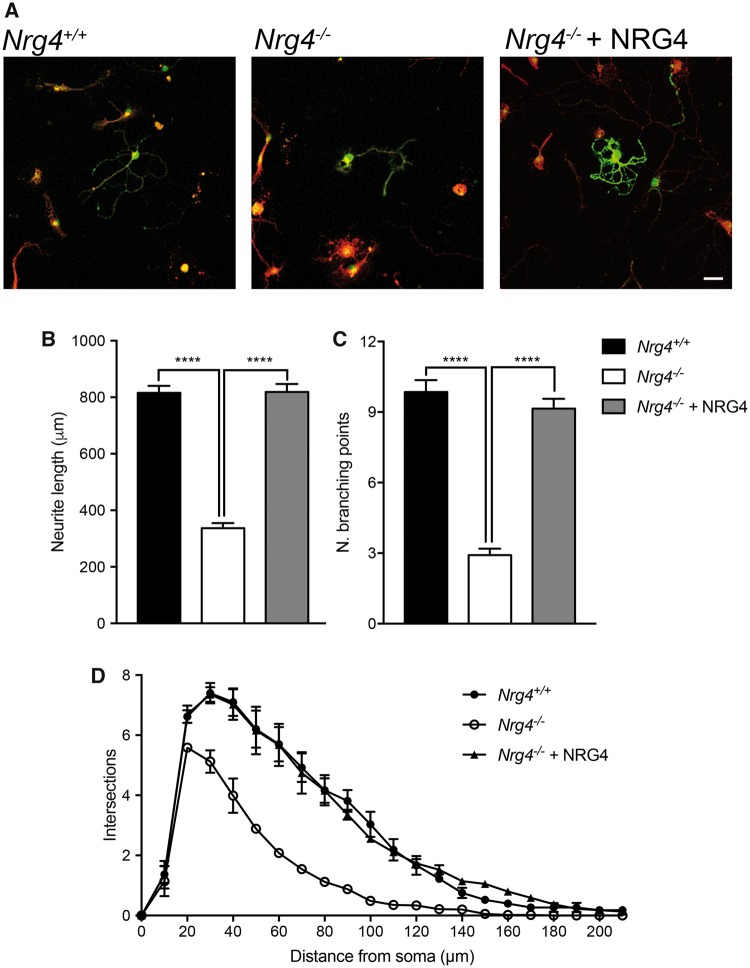
The phenotype of striatal MSNs of *Nrg4^−^*^/^^*−*^ mice is replicated in culture and is rescued by recombinant NRG4. **(A)** Representative photomicrographs of striatal MSNs of E14 *Nrg4*^+/+^ and *Nrg4^−^*^/^^*−*^ mice cultured for 10 days. Cultures were supplemented with 100 ng/mL recombinant NRG4 at plating, as indicated. Neurons were double labeled for βIII-tubulin (red) and DARPP-32 (green) to identify MSNs. Scale bar = 25 μm. Total neurite length **(B)**, branch point number **(C)**, and Sholl plots **(D)** of MSNs in these cultures are shown. Mean ± SEM of data collected from 3 independent experiments (****p < 0.0001, one-way ANOVA with Tukey’s multiple comparison post hoc test).

Similar to the phenotypic changes observed in Golgi preparations of *Nrg4*^*−*^^/^^*−*^ mice, the neurite arbors of MSNs cultured from *Nrg4*^*−*^^/^^*−*^ mice were smaller and less complex than those of MSNs cultured from *Nrg4*^+/+^ littermates (Fig. 5A). Measurement of total neurite length and branch point number confirmed highly significant 2- to 3-fold decreases in neurons cultured from *Nrg4*^*−*^^/^^*−*^ mice compared with those cultured from *Nrg4*^+/+^ littermates (Fig. 5B, C). The differences in the size and complexity of neurite arbors of neurons cultured from *Nrg4*^*−*^^/^^*−*^ and *Nrg4*^+/+^ littermates were reflected in the Sholl plots of these neurons (Fig. 5D), which provide graphic representation of neurite length and branching with distance from the cell body. The reductions in neurite length and branching in DARPP-32-positive MSNs cultured from *Nrg4*^*−*^^/^^*−*^ mice were completely restored to wild type levels by treatment with recombinant NRG4 (Fig. 5). These results show that the MSN phenotype observed in vivo in NRG4-deficient mice is replicated in cultured neurons and is completely rescued by soluble recombinant NRG4 treatment.

MSNs cultured from wild type embryos responded with increased neurite growth and branching to NRG4 in a dose-dependent manner (Supplementary Data Fig. S1). This suggests that endogenous synthesis of NRG4, at least in culture, is not saturating.

### MSNs Coexpress NRG4 and ErbB4

**FIGURE 6.**
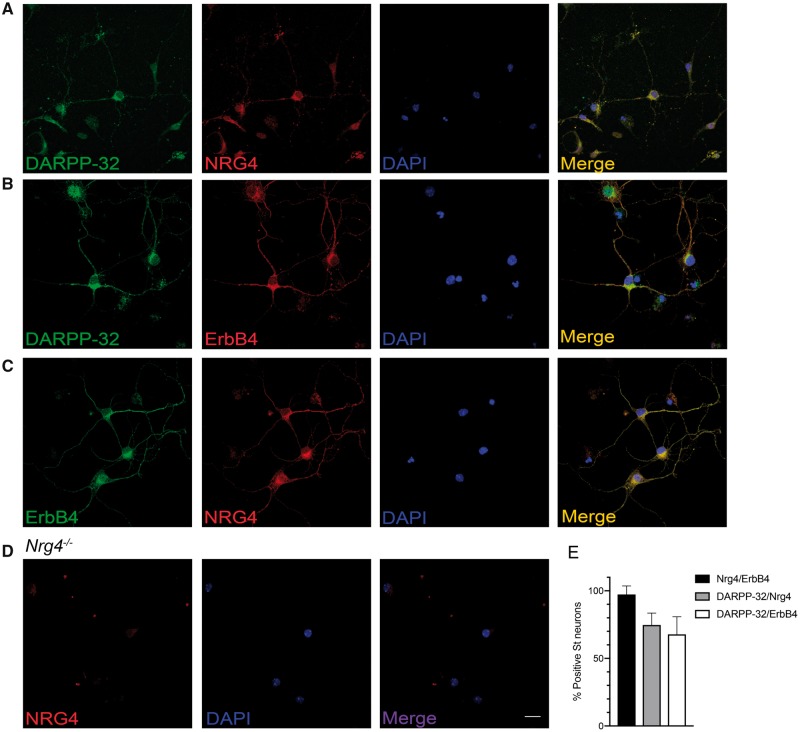
Cultured striatal MSNs coexpress NRG4 and ErbB4. **(A–C)** Representative photomicrographs of MSNs of E14 embryos cultured for 10 days and double labeled with antibodies to DARPP-32 and NRG4 **(A)**, DARPP-32 and ErbB4 **(B)**, or NRG4 and ErbB4 **(C)**. To demonstrate the specificity of the anti-NRG4 antibody, cultures were set up from E14 *Nrg4^−^*^/^^*−*^ embryos, in which NRG4 immunoreactivity was not observed **(D)**. To identify nuclei, all cultures were counterstained with DAPI. Scale bar = 20 μm. **(E)** Quantification of the number of positive neurons for NRG4 and ErbB4, DARPP-32 and NRG4, and DARPP-32 and ErbB4. Data are % positive neurons ± SEM from least 90 neurons collected from 3 independent experiments.

To ascertain the identity of the cells that produce NRG4 in striatum, we studied NRG4 immunofluorescence in dissociated MSN cultures. Double labeling for NRG4 and the specific MSN marker DARPP-32 revealed that 74.72% ± 8.7% of the DARPP-32-positive cells were colabeled with anti-NRG4 (Fig. 6A, E). In double labeling experiments using anti-DARPP-32 and anti-ErbB4, 67.82% ± 13% of the DARPP-32-positive cells were also colabeled with anti-ErbB4 (Fig. 6B, E). These observations suggest that most MSNs coexpress NRG4 and ErbB4. To formally demonstrate coexpression, we double labeled MSN cultures with antibodies to NRG4 and ErbB4. Accordingly, 97.33% ± 6.26% of neurons that were immunopositive for NRG4 were also immunopositive for ErbB4 (Fig. 6C). NRG4 immunoreactivity was not observed in cultures established from *Nrg4*^*−*^^/^^*−*^ mice, demonstrating the specificity of the anti-NRG4 antibody (Fig. 6D). Taken together, these findings suggest that most MSNs coexpress NRG4 and ErbB4 and raise the possibility that NRG4 exerts its effects on MSNs by an autocrine/paracrine mechanism.

While immunohistochemical expression of ErbB4 in striatum has been reported (28), we also demonstrated NRG4 immunolabeling and overlapping DARPP-32 immunolabeling in sections of the striatum, which provides an additional demonstration of NRG4 protein expression in the striatum (Supplementary Data Fig. S2). NRG4 immunolabeling was absent in the striatum of *Nrg4*^*−*^^/^^*−*^ mice, further demonstrating the specificity of the anti-NRG4 antibody.

## DISCUSSION

Striatal MSNs comprise a large, clinically important population of neurons that are extensively connected with several major brain regions. Characteristic changes in the exuberant dendritic arbors and spine density of these neurons have been described in many neurological conditions and are also caused by a variety of recreational and medicinal drugs (2, 4–10). Despite this wealth of observational data, the physiological regulators of the growth and elaboration of MSN dendrites and the regulators of dendritic spine formation on these neurons remain poorly understood. Here, we report that NRG4 is a major novel, physiologically relevant regulator of MSN dendrite growth and elaboration and MSN dendrite spine formation and maturation. We have shown that neonatal mice that lack NRG4 have markedly stunted MSN dendritic arbors compared with wild type littermates. NRG4-deficient neonates displayed highly significant reductions in total dendrite length and in dendrite branching. While dendrite arbor size and complexity could not be clearly quantified at later stages, a limitation of this aspect of our study, juvenile and adult NRG4-deficient mice nonetheless displayed a highly significant reduction in MSN dendrite spine number per unit dendrite length compared with wild type mice. This significant decrease was already evident in NRG4-deficient neonates, showing that it arises in development and remains substantially unchanged as nervous system matures. In addition to the marked reduction in the number of MSN dendrite spines in *Nrg4*^*−*^^/^^*−*^ mice, there was a significant reduction in the proportion of spines that exhibited a mature morphology in juvenile and adult *Nrg4*^*−*^^/^^*−*^ mice. These results show that NRG4 plays an important physiological role in contributing to the formation and maturation of MSN dendrite spines.

The neuregulin family of secreted growth factors has a multitude of functions in a variety of tissues and has a particularly extensive repertoire of roles in the nervous system in development, health and disease (16–22). NRG4 is atypical in that it is expressed in a limited number of adult tissues and has been reported to have negligible expression in adult brain compared with other neuregulins (29, 30). Perhaps because of this, its potential neural functions have received little attention. Indeed, only one function has been described for NRG4 in the brain, that of promoting the growth and elaboration of pyramidal dendrites in the developing mouse neocortex (23). However, it has yet to be investigated whether cortical pyramidal neurons in *Nrg4*^*−*^^/^^*−*^ mice, like MSNs, also have a deficit in dendritic spines. In the peripheral nervous system, it has recently been reported that NRG4 is a physiological regulator of sympathetic axon growth and regulates adipose tissue innervation (31).

The marked dendrite phenotype of MSNs observed in *Nrg4*^*−*^^/^^*−*^ mice could result from either the loss of a direct action of endogenous NRG4 on MSNs or an indirect effect of the absence of NRG4. Several observations suggest that NRG4 acts directly on developing MSNs. First, we have shown that most MSNs express ErbB4, the principal NRG4 receptor tyrosine kinase, and thus have the capacity to respond to NRG4 directly. Second, in dissociated culture, in which MSNs no longer retain their characteristic rich connections with other neurons, they nonetheless extend shorter, less branched neurites when cultured from *Nrg4*^*−*^^/^^*−*^ embryos, compared with *Nrg4*^+/+^ embryos. This suggests that the stunted neurite arbors of MSNs cultured from NRG4-deficient embryos is not dependent on their connections with other neurons. Third, the stunted neurites of MSNs cultured from *Nrg4*^*−*^^/^^*−*^ embryos can be restored in size and complexity to those of MSNs cultured from *Nrg4*^+/+^ embryos by NRG4 treatment. This not only suggests that MSNs are able to respond to NRG4 directly but also suggests that no indirect effect of NRG4 is needed to account for the stunted in vivo dendrite phenotype of MSNs of *Nrg4*^*−*^^/^^*−*^ mice since the rescue of the in vitro phenotype is complete.

The coexpression of NRG4 and ErbB4 on MSNs, at least in culture, raises the possibility that NRG4 exerts its effects on MSNs in vivo by an autocrine mechanism. Likewise, neocortical pyramidal neurons coexpress NRG4 and ErbB4, raising the possibility that NRG4 acts on these neurons by an autocrine route (23). NRG1 autocrine signaling promotes remyelination following peripheral nerve injury (32), and an ErbB4/NRG2 autocrine signaling loop has been reported in inhibitory interneurons (33). Neuregulin autocrine signaling has also been implicated outside the nervous system, for example, in promoting the proliferation of certain cancers (34).

In addition to autocrine NRG4, MSNs may also obtain NRG4 from the neurons with which they synapse. MSNs project to globus pallidus and midbrain (2), and the report that *Nrg4* mRNA is expressed in the newborn midbrain (23) raises the possibility that MSNs may obtain NRG4 by a retrograde paracrine route from this target. MSNs receive afferents from several brain regions (1), and the report that *Nrg4* mRNA is detectable in the cerebral cortex of newborn mice (23) raises the possibility that MSNs may obtain NRG4 by an anterograde paracrine route. There is, for example, evidence that MSNs may obtain BDNF from the cerebral cortex by an anterograde route (35).

In summary, our in vivo studies of striatal MSNs show that NRG4 is a prominent novel physiological regulator of the growth and elaboration of MSN dendrites and the formation and maturation of dendritic spines. Furthermore, our in vitro studies suggest that NRG4 acts directly on MSNs during development. This work raises several important questions for future research, namely, whether the striking morphological phenotype of MSNs in NRG4-deficient mice is accompanied by a behavioral phenotype and whether there are additional functional changes in NRG4-deficient MSNs that are not directly related to the morphological phenotype.

## Supplementary Material

Supplement_Material_nlz046

## Notes

This work was supported by Wellcome Trust grant 103852 to Alun M. Davies. Blanca Paramo was supported by a fellowship from CONACyT number 711083.
