# Supplementary material for: Neuregulin-4 Is Required for the Growth and Elaboration of Striatal Medium Spiny Neuron Dendrites
Source: J Neuropathol Exp Neurol. 2019 May 24;78(8):725–34. doi: 10.1093/jnen/nlz046 (PMC6640913; doi:10.1093/jnen/nlz046)
Supplement: Supplement_Material_nlz046 [file supplement_material_nlz046.pdf]

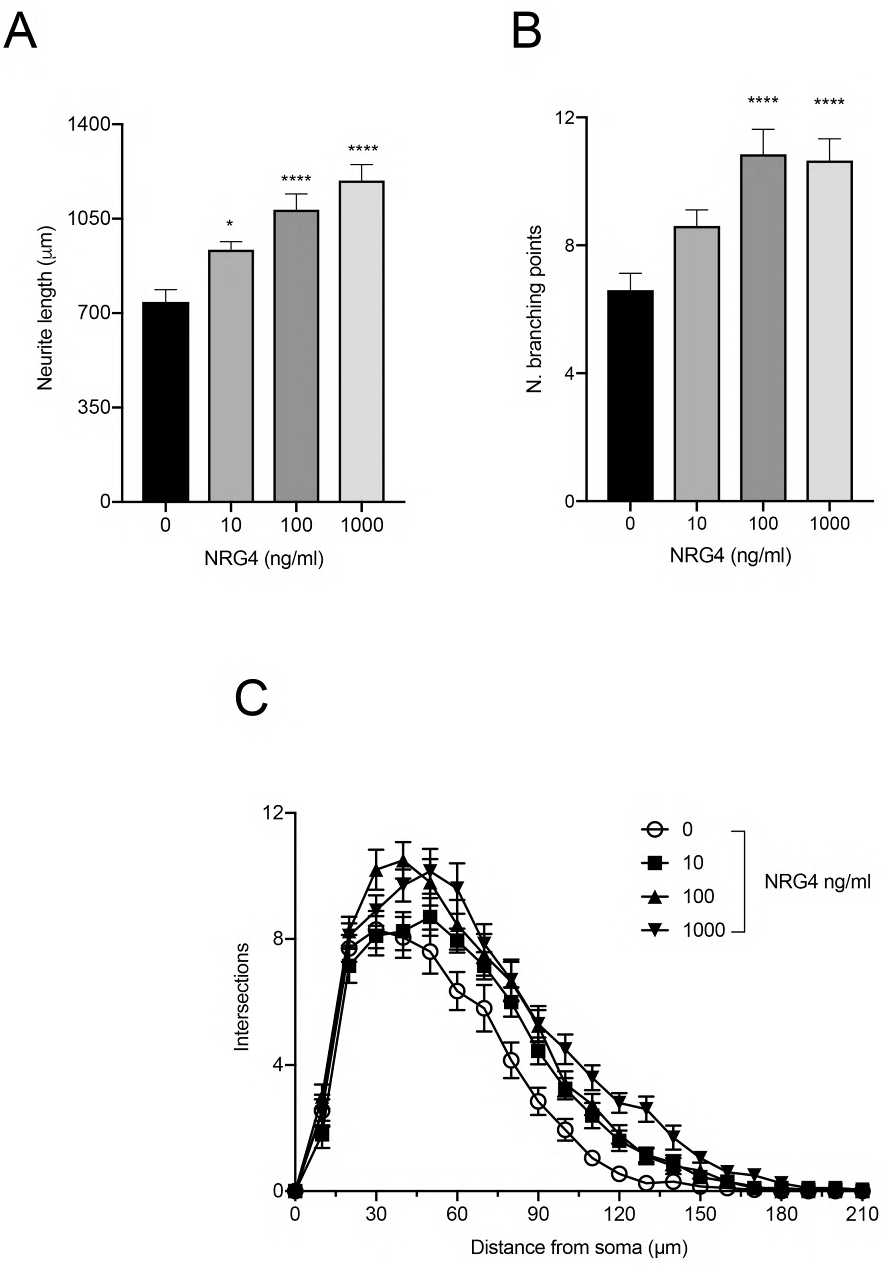

Figure S1. NRG4 enhances dendrite growth of cultured medium spiny neurons. Quantification of total dendrite length (**A**), number of branch points (**B**) and Sholl analysis (**C**) of MSN after 10 days in culture from *Nrg4*$^{+/+}$ mice treated with NRG4 at different concentrations. The mean ± s.e.m. of data from 20 neurons per condition from independent experiments are plotted (*P<0.05, ****P < 0.001, statistical comparison versus no treatment, Dunnett's t-test).

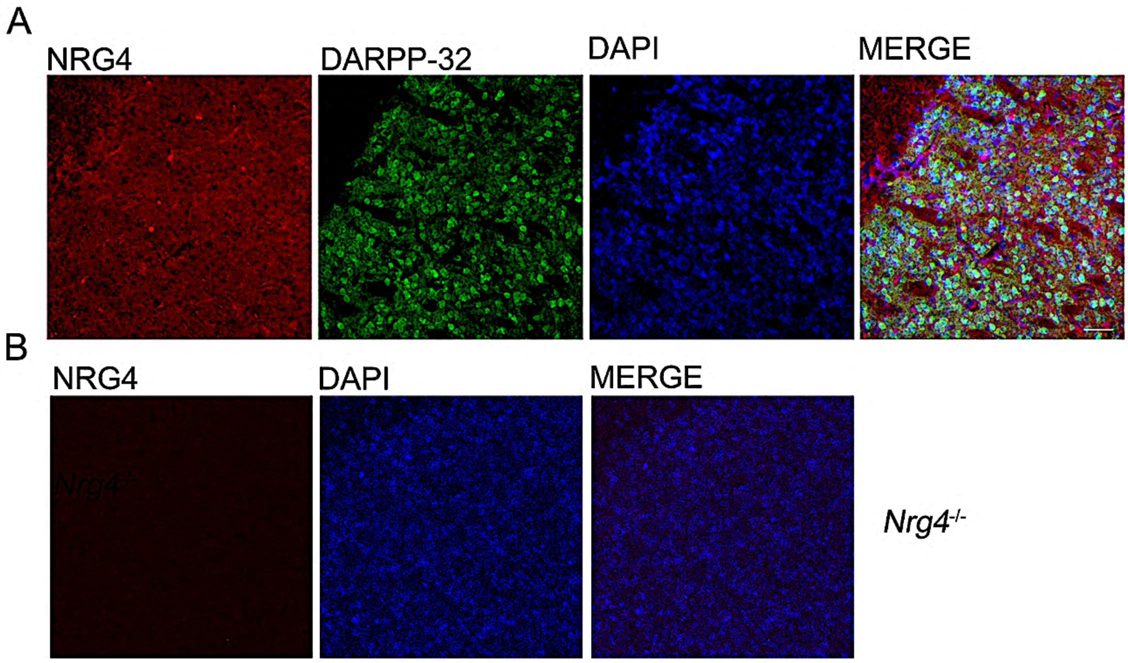

Figure S2. Developing striatal neurons co-express NRG4 and DARPP-32. (**A**) Representative sections of the striatum of P10 mice double labelled with antibodies to NRG4 and DARPP-32. Scale bar = 100 μm. (**B**) To demonstrate the specificity of the anti-NRG4 antibody, sections from *Nrg4*$^{-/-}$ mice were stained with anti-NRG4 and anti-DAPI.
